# Enhanced production of ginsenoside compound K by synergistic conversion of fermentation with *Aspergillus tubingensis* and commercial cellulase

**DOI:** 10.3389/fbioe.2024.1538031

**Published:** 2025-01-08

**Authors:** Yong-In Lee, Woo-Seok Song, Deok-Kun Oh

**Affiliations:** Department of Bioscience and Biotechnology, Konkuk University, Seoul, Republic of Korea

**Keywords:** *Aspergillus tubingensis*, commercial cellulase, synergistic conversion, compound K, ginseng extract, fermentation, enzyme conversion

## Abstract

Fermentation of ginseng extract is limited by the low concentration of compound K (CK), a bioactive ginsenoside. In this study, a novel approach combining *Aspergillus tubingensis* fermentation with *Aspergillus niger* cellulase conversion was used to enhance CK production from high concentrations of American ginseng extract (AGE). The reaction conditions, including the feeding rate and concentrations of carbon source, enzyme type, AGE and enzyme concentrations, temperature, pH, and timing of enzyme addition, were optimized. Under optimized conditions, this combined method achieved an enhanced CK production of 8.06 g/L (13.0 mM) after 168 h, with a productivity of 48 mg/L/h. This approach led to a 2.0-fold increase in concentration and a 1.7-fold increase in productivity when compared with traditional fermentation using the same strain. The findings of this study demonstrate the synergistic effect of combining fermentation with enzyme conversion to improve CK production.

## 1 Introduction

Ginseng (*Panax ginseng* C.A. Meyer) has been used as a traditional medicine in East Asia for over a thousand years to enhance immunity and maintain physical vitality (Yang et al., 2015). The pharmacological activity of ginseng is primarily attributed to ginsenosides, which are classified into two main types: protopanaxadiol (PPD) and protopanaxatriol. PPD-type ginsenosides possess one hydroxyl group at C-12, with glycosides at C-3 and C-20 on dammarane-type tetracyclic triterpenoid aglycones, whereas protopanaxatriol-type ginsenosides contain two hydroxyl groups at C-3 and C-12, with glycosides at C-6 and C-20 (Park et al., 2010).

Major ginsenosides, such as Rb1, Rb2, Rc, and Rd, which contain three to four glycoside molecules, comprise more than 80% of the total ginsenosides found in natural ginseng. In contrast, minor ginsenosides such as F2, Rg3, compound Y (CY), compound Mc (CMc), and compound K (CK), which have one to two glycoside molecules, are either present in very low quantities or absent in natural ginseng and are produced by the deglycosylation of major ginsenosides. Minor ginsenosides generally exhibit higher pharmacological activity than major ginsenosides because of their enhanced absorption due to their superior permeability in the gastrointestinal tract. CK, 20-O-β-D-glucopyranosyl-20(S)-PPD, is one of the most bioactive ginsenosides, and demonstrates a range of beneficial effects, including anti-oxidative, anti-diabetic, anti-inflammatory, anti-cancer, and hepatoprotective activities (Li et al., 2012; Igami et al., 2015; Quan et al., 2015; Hossen et al., 2017; Yin et al., 2021). Consequently, CK has been widely employed in the food, cosmetic, and pharmaceutical industries owing to its excellent pharmacological activities.

CK can be produced by the deglycosylation of PPD-type major ginsenosides using biological methods, such as whole cell, enzyme, and fermentation conversions (Shin and Oh, 2016). Whole-cell conversion is limited by low gene expression levels within cells and the hindrance posed by cell membranes to substrate-enzyme interactions (Kim et al., 2019). Enzyme conversion generally offers higher concentrations and productivity than fermentation. However, it requires cell harvesting and enzyme purification, leading to increased costs (Jeong et al., 2020). In contrast, fermentation is a cost-effective process that does not require these additional steps. Both intracellular and extracellular enzymes can be used in this process, although it often results in lower concentration and productivity (Han et al., 2007; Zhou et al., 2008; Bae et al., 2011; Hsu et al., 2013; Li et al., 2016). Recent studies on traditional fermentation have focused on enhancing the concentration and productivity of CK by optimizing the feeding methods of carbon source and ginseng extract during fermentation (Song et al., 2022; Song et al., 2023). However, further improvements in the concentration and productivity of CK are essential for industrial production, indicating that a novel method to overcome the limitations of traditional fermentation is needed.

In this study, to enhance the bioconversion of American ginseng extract (AGE) into CK, a food-grade commercial enzyme was added following fermentation with *Aspergillus tubingensis*, which is a generally recognized as safe (GRAS) microorganism. The reaction conditions were optimized, focusing on the feeding rate and concentrations of carbon source during the cultivation phase as well as temperature, pH, addition timing of enzyme, and concentrations of AGE and commercial enzyme during the enzyme conversion phase. Under these optimized conditions, enhanced CK concentration and productivity were achieved by synergistic conversion of enzymes derived from cultivation and commercial enzyme.

## 2 Materials and methods

### 2.1 Materials

Ginsenoside standards (≥98% purity), including Rb1, Rc, Rb2, Rd, F2, compound Mc1 (CMc1), compound O (CO), CMc, CY, and CK, were purchased from Ambo Institute (Seoul, Republic of Korea). AGE was purchased from Ace EMzyme (Ansung, Republic of Korea) and contained 38.5% (w/w) PPD-type ginsenosides, specifically, 20.4% Rb1, 1.2% Rb2, 7.4% Rc, and 9.5% Rd. Commercial enzymes, Cellulosin AL8 (cellulase) and Pluszyme 2000P (β-glucosidase) from *Aspergillus niger*, were purchased from Bision Corporation (Seoul, Republic of Korea). Commercial enzymes, Pectinex Ultra SP-L (pectinase) and Viscozyme L (mixture of β-glucanase, pectinase, hemicellulose, and xylanase) from *Aspergillus aculeatus* were obtained from Novozymes (Copenhagen, Denmark).

### 2.2 Fungal strain and culture media

*Aspergillus tubingensis* [Korean Collection for Type Cultures, (KCTC) 14,166, Daejeon, Republic of Korea] was used to produce CK from PPD-type ginsenosides in AGE. Fermentation medium consisted of 10 g/L sucrose, 10 g/L soy protein concentrate, 2 g/L rice straw, 5 g/L KH2PO4, 5 g/L Na2HPO4, 0.3 g/L MgSO4·7H2O, 0.3 g/L CaCl2, 5 mg/L FeSO4·7H2O, and 1.3 mg/L MnSO4·H2O.

### 2.3 Culture conditions

The inoculum preparation of *A. tubingensis* was conducted as previously described (Song et al., 2022). For flask experiments, the mycelia were then transferred to a 250 mL baffled flask containing 50 mL of fermentation medium, which was incubated at 28°C with agitation at 150 rpm for 60 h to prepare the fermentation broth used for enzyme conversion or 144 h for CK production by fermentation.

For the fermenter experiments, the mycelia were incubated at 28°C for 24 h in a 250-mL baffled flask and transferred to a 3 L fermenter (BioCNS, Daejeon, Republic of Korea) containing 1 L of fermentation medium. Cultivation was performed by maintaining a pH of 5.0 and a temperature of 28°C, with an aeration rate of 1 L/min. The agitation speed was adjusted from 150 to 1,000 rpm to maintain the dissolved oxygen levels above 20% during the cultivation phase (Song et al., 2023).

### 2.4 CK production by fermentation and/or enzyme conversion

Fermentation of *A. tubingensis* was performed at 28°C for 144 h by adding 16 or 40 g/L AGE to the fermentation broth at 60 h. Enzyme conversion was conducted at 60°C in 50 mM citrate/phosphate buffer (pH 5.0) containing 0.5 or 1.25 mg/mL *A. niger* cellulase (Cellulosin AL8) and 16 or 40 g/L AGE for 84 h. After cultivation of *A. tubingensis* at 28°C for 60 h, 16 or 40 g/L AGE was added to the fermentation broth at 60 h. The resulting mixture was incubated at 60°C for an additional 84 h without or with the addition of Cellulosin AL8. For enzyme selection, 16 g/L AGE and 0.4 mg/mL commercial enzyme were added to the fermentation broth at 60 h.

### 2.5 Ginsenoside specificity

For enzyme preparation, the broth of *A. tubingensis* following fermentation was filtered, and four volumes of methanol were added to the filtrate. The precipitate obtained by centrifugation at 13,000 × g at 4°C for 20 min after overnight incubation at 4°C was dissolved in 50 mM citrate/phosphate buffer (pH 5.0) and dialyzed using a dialysis tube. The dialyzed solution was then filtered by ultrafiltration using a Centricon with a 10 kDa cut-off size (Amicon Ultra-15, Millipore, United States). The filtered solution was used as extracellular enzymes from *A. tubingensis* to evaluate the ginsenoside specificity.

The specific activities of extracellular enzymes from *A. tubingensis* and cellulase from *A. niger* towards PPD-type ginsenosides were determined by measuring the decrease in the amount of reactant ginsenoside. The reactions were performed at 60°C for 10 min in 50 mM citrate/phosphate buffer (pH 5.0) containing 0.4 mg/mL each ginsenoside, by adjusting the enzyme concentration from 0.0001 to 0.1 mg/mL. One unit (U) of enzyme activity was defined as the amount of enzyme required to decrease 1 nmol of reactant ginsenoside per min.

### 2.6 Optimization of temperature-shift time and temperature

The reaction temperature of *A. tubingensis* was changed from 28°C to 60°C, with temperature-shift times ranging from 48 to 96 h, to determine the optimal temperature-shift time from the cultivation phase to the enzyme conversion phase. At each temperature shift time, 0.4 mg/mL *A. niger* cellulase and 16 g/L AGE were added to the fermentation broth, which was then reacted at 60°C for an additional 84 h. After the cultivation of *A. tubingensis* at 28°C for 60 h, the reactions were performed for an additional 84 h by varying the temperature from 50°C to 70°C.

### 2.7 Optimization of AGE and commercial enzyme concentrations

After cultivation for 60 h, the reactions were conducted at 60°C for an additional 84 h by varying the concentration of AGE from 4 to 48 g/L with 1.0 mg/mL *A. niger* cellulase, the concentration of *A. niger* cellulase from 0.1 to 1.4 mg/mL with 32 g/L AGE, and the concentrations of AGE and *A. niger* cellulase at an optimal ratio of 32:1 (w/w) ranging from 8 to 0.25–64 g/L and 2.0 mg/mL, respectively.

### 2.8 Optimization of AGE feeding and temperature-shift time in a fermenter

The method of feeding AGE in fermenter was determined by comparing pulse feeding and continuous feeding. Pulse feeding was performed by adding 40 g/L AGE into the fermenter at 60 h, while continuous feeding was conducted by supplementing 40 g/L AGE from 12 to 84 h using a peristaltic pump at a feeding rate of 0.56 g/L/h.

The reaction temperature of *A. tubingensis* was changed from 28°C to 60°C, with temperature-shift times ranging from 36 to 60 h. An initial sucrose concentration of 20 g/L was added, followed by continuous feeding of 12 g/L sucrose into the fermenter from 12 to 36, 48, 54, and 60 h at feeding rates of 0.50, 0.33, 0.29, and 0.5 g/L/h, respectively. *A. niger* cellulase at 1.25 mg/mL was added at each temperature shift, whereas 40 g/L AGE was continuously added to the fermenter from 12 to 84 h at a feeding rate of 0.56 g/L/h. The total reaction time, which included both the cultivation and enzyme conversion times, was 168 h.

### 2.9 Optimization of sucrose feedings in a fermenter

The effect of added sucrose concentration on CK production was evaluated by varying the concentration from 6 to 18 g/L, with an initial sucrose concentration of 20 g/L. Sucrose concentrations of 6, 12, and 18 g/L were fed from 12 to 48 h at rates of 0.17, 0.33, and 0.50 g/L/h, respectively.

At an added sucrose concentration of 12 g/L, sucrose concentrations of 10, 11, 11.5, and 12 g/L sucrose were fed by varying the period of major feeding from 12 to 24, 36, 42, and 48 h at rates of 0.83, 0.46, 0.38, and 0.33 g/L/h, respectively, to determine the optimal major feeding period. Following this, residual sucrose concentrations of 2.0, 1.0, and 0.5 g/L were fed from 24, 36, and 42–48 h, respectively, at a rate 0.083 g/L/h as activity-maintaining feeding, which was the minimal feeding rate required to maintain the CK-producing activity of *A. tubingensis* during the period excluding the major feeding period (Song et al., 2023) 17.

### 2.10 Optimization of pH and temperature in a fermenter

An initial sucrose concentration of 20 g/L was added, followed by continuous feeding of sucrose concentrations of 11 and 1.0 g/L from 12 to 36 h at a rate of 0.46 g/L/h, and from 36 to 48 h at a rate of 0.083 g/L/h, respectively. An AGE concentration of 40 g/L was continuously supplied from 12 to 84 h at a rate of 0.56 g/L/h, while 1.25 mg/mL *A. niger* cellulase was added at 48 h. During the enzyme conversion phase, the reactions were conducted at an agitation speed of 200 rpm without aeration by varying the pH from 4.0 to 5.0 at a constant temperature of 60°C, and the temperature was changed from 50°C to 60°C at a constant pH of 4.5.

### 2.11 Analytical methods

After termination, the reaction mixture was extracted using an equal volume of n-butanol. The extracted solution was supplemented with 1.0 mg/mL digoxin as an internal standard. The n-butanol layer was collected, evaporated to dryness, and the dried residue was dissolved in methanol. The concentrations of ginsenosides in the methanol solution were analyzed using a high-performance liquid chromatography system (Agilent 1,100; Santa Clara, CA, United States) equipped with an ultraviolet detector set at 203 nm and octadecyl-silica column (YMC, Kyoto, Japan). The column was eluted at a flow rate of 1 mL/min at 40°C using a gradient of acetonitrile/water (v/v) as follows: 30:70 to 60:40 for 20 min, 60:40 to 90:10 for 10 min, 90:10 to 30:70 for 5 min, and finally maintained at 30:70 for 10 min.

## 3 Results and discussion

### 3.1 Selection of commercial enzyme for reducing accumulated precursor ginsenosides in CK production by fermentation

**FIGURE 1:**
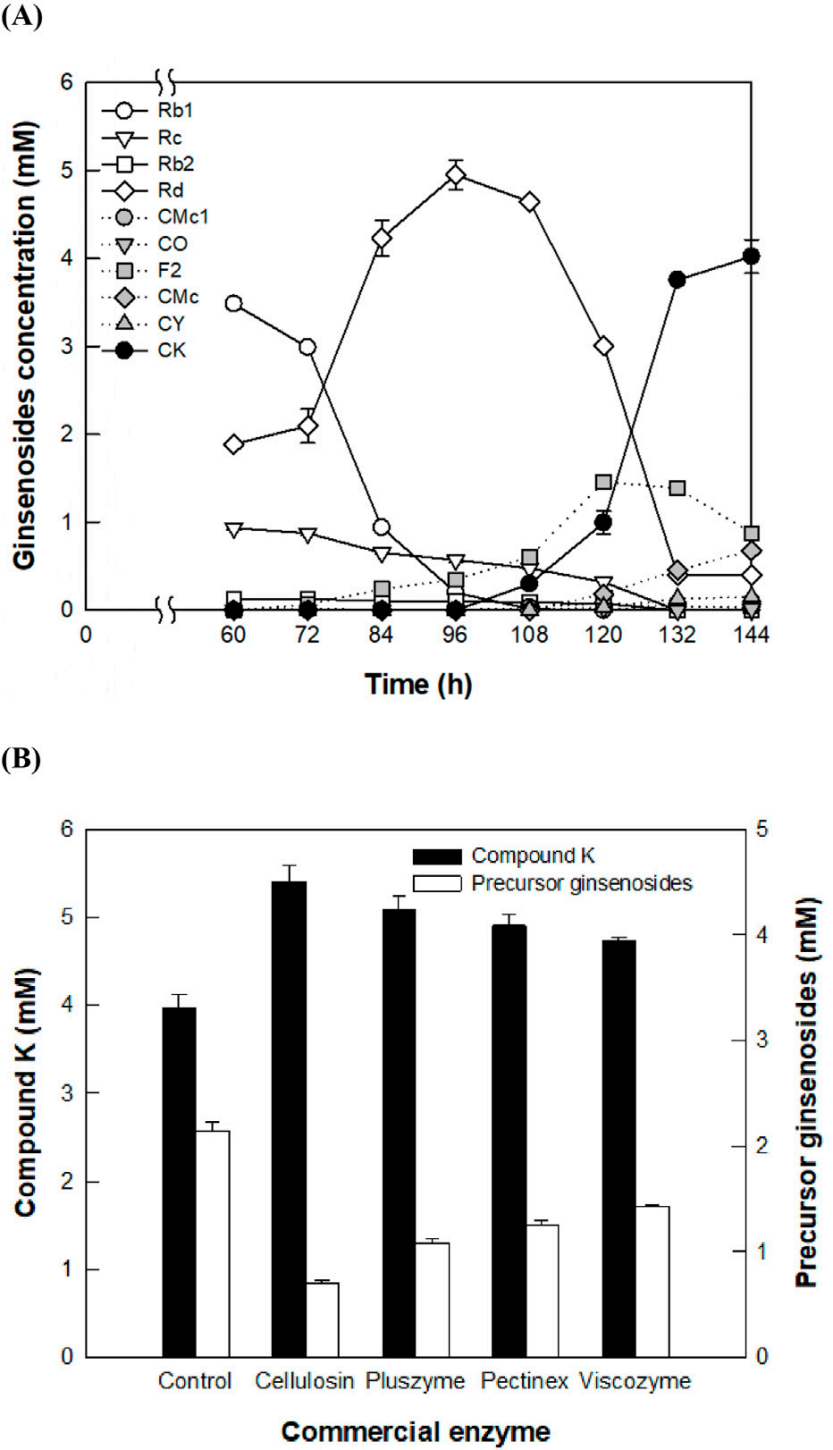
Reduction of accumulated precursor ginsenosides in the production of compound K (CK) during *Aspergillus tubingensis* fermentation by the addition of commercial enzymes. **(A)** Accumulation of precursor ginsenosides during the production of CK from protopanaxadiol (PPD)-type ginsenosides in American ginseng extract (AGE) by fermentation. Fermentation was performed at 28°C for 144 h by adding 16 g/L AGE to the culture broth at 60 h. **(B)** Enhancement of CK production and reduction of precursor ginsenosides by the addition of commercial enzymes. Each commercial enzyme was added at a concentration of 0.4 mg/mL at 60 h of *A. tubingensis* cultivation, and the mixtures were reacted at 60°C for an additional 84 h. The control did not involve the addition of commercial enzyme.

The concentration of CK produced from PPD-type ginsenosides in 16 g/L AGE by fermentation of *A. tubingensis* at 28°C for 144 h was 4.0 mM (2.51 g/L) (Figure 1A). Precursor ginsenosides, including Rd, F2, CMc, CMc1, CO, and CY, accumulated to 2.14 mM after 144 h of fermentation, with Rd reaching a concentration of 5.0 mM at 96 h.

The highest accumulation of Rd in the fermentation of *A. tubingensis* was achieved at the middle reaction time. AGE contains 53.0% Rb1, 24.7% Rd 19.2% Rc, and 3.1% Rb2 in PPD-type ginsenosides. Shortly after the addition of AGE, the sugar moieties attached to the C20 position of Rb1, Rc, and Rb2 were hydrolyzed, resulting in the production of Rd. The extracellular enzymes from *A. tubingensis* exhibited significantly higher specific activity toward Rb1 compared to other PPD-type ginsenosides (Table 1), indicating that the main pathway of PPD-type ginsenosides to form CK follows Rb1 → Rd → F2 → CK. The specific activity of these extracellular enzymes for the conversion of Rb1 into Rd was 6.9-fold higher than that for the conversion of Rd into F2. Therefore, the observed accumulation of Rd in the fermentation can be attributed to the relative abundance of Rb1 and Rd in AGE, the highest activity toward Rb1, and lower activity toward Rd.

**TABLE 1:** Specific activity of extracellular enzymes from *A. tubingensis* and cellulase from *A. niger* for PPD-type ginsenosides.

Substrate^a^	Specific activity (U/mg)	
	Extracellular enzymes from *Aspergillus tubingensis*	Cellulase from *Aspergillus niger*
Rb1	1765 ± 43	3,030 ± 52
Rb2	103 ± 5	255 ± 9
Rc	760 ± 24	215 ± 8
Rd	255 ± 8	630 ± 21
Compound Mc1	132 ± 8	166 ± 9
Compound O	289 ± 9	83 ± 3.9
F2	103 ± 5	23 ± 1.8
Compound Mc	35 ± 1.9	9.0 ± 1.6
Compound Y	18 ± 0.4	56 ± 1.9
Compound K	0.5 ± 0.03	0.0 ± 0.0

Data represents the means of duplicate experiments, and error bars are expressed as standard deviation.

^a^ Substrate concentration was 0.4 mM.

Reducing the accumulation of precursor ginsenosides through fermentation alone is a time-consuming and inefficient process. Therefore, a method involving the addition of commercial enzymes to reduce precursor ginsenosides was used. When a commercial enzyme such as Cellolosin AL8, Pectinex SP-L, Pluszyme 2000P, or Viscozyme L was added to the fermentation broth, the concentration of precursor ginsenosides decreased because of the deglycosylation of *A. tubingensis* fermentation and commercial enzymes, resulting in an increase in CK production. Among the four commercial enzymes tested, Cellolosin AL8 (*A. niger* cellulase) exhibited the highest concentration of CK (5.40 mM) and the lowest concentration of precursor ginsenosides (0.70 mM) (Figure 1B). Therefore, *A. niger* cellulase was selected to enhance CK production.

### 3.2 Comparison of CK production by fermentation with *A. tubigensis* and/or enzyme conversion using commercial cellulase

CK production from 16 g/L AGE was assessed using the following four methods. CK was produced at a concentration of 4.0 mM by fermentation with *A. tubingensis* at 28°C for 144 h (Figure 1A) and enzyme conversion using *A. niger* cellulase at 60°C for 84 h (Supplementary Figure S1A). *A. tubingensis* was cultivated at 28°C for 60 h, after which the temperature was increased from 28°C to 60°C. The fermentation broths of *A. tubingensis* without and with the addition of *A. niger* cellulase at 60 h produced 4.4 mM and 5.7 mM of CK for an additional 84 h, respectively (Supplementary Figures S1B, C). Thus, the highest CK production was obtained by the combination of fermentation and enzyme conversion.

**FIGURE 2:**
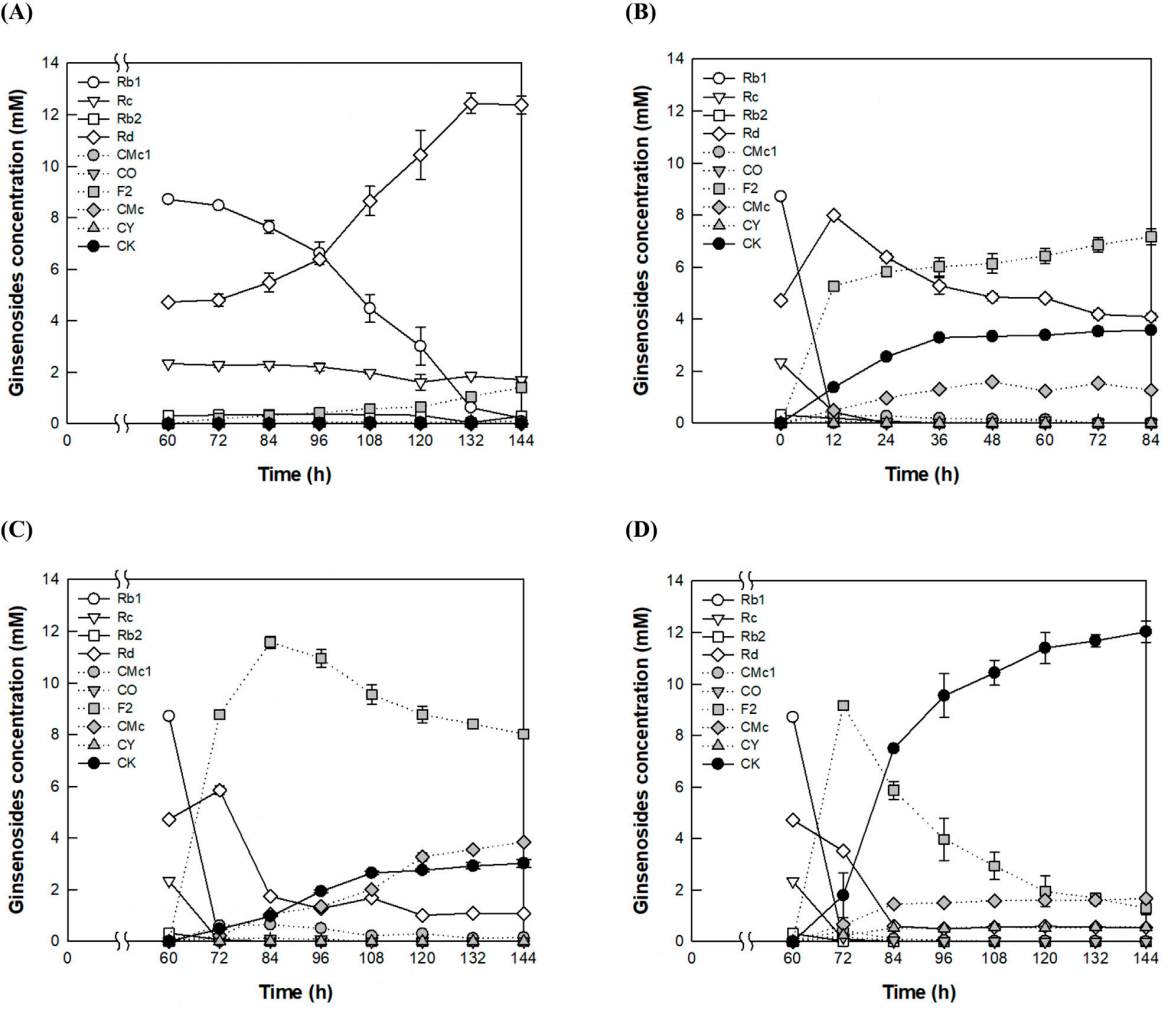
Synergistic production of CK from PPD-type ginsenosides in AGE by fermentation and enzyme conversion. **(A)** CK production by fermentation of *A. tubingensis*. The fungus was cultivated at 28°C for 60 h, followed by the addition of 40 g/L AGE at 60 h, and the fermentation was carried out at 28°C for 144 h. **(B)** CK production by enzyme conversion of *A. niger* cellulase. The reaction was performed at 60°C in 50 mM citrate/phosphate buffer (pH 5.0) containing 40 g/L AGE and 1.25 mg/mL *A. niger* cellulase for 84 h. **(C)** CK production by enzyme conversion of the fermentation broth derived from *A. tubingensis* cultivation. The fungus was cultivated at 28°C for 60 h, followed by the addition of 40 g/L AGE at 60 h, and the reaction was performed at 60°C for an additional 84 h. **(D)** CK production by the addition of *A. niger* cellulase to the fermentation broth of *A. tubingensis*. The fungus was cultivated at 28°C for 60 h, after which 40 g/L AGE and 1.25 mg/mL *A. niger* cellulase were added at 60 h, and the reaction was performed at 60°C for an additional 84 h.

CK production at a high concentration of 40 g/L AGE was also evaluated using these four methods. No CK was produced by fermentation with *A. tubingensis* at 28°C for 144 h (Figure 2A), whereas CK was produced at a concentration of 3.6 mM by enzyme conversion with *A. niger* cellulase at 60°C for 84 h (Figure 2B). After cultivation of *A. tubingensis* at 28°C for 60 h, the fermentation broth at 60°C for an additional 84 h produced 3.0 mM CK (Figure 2C). When *A. niger* cellulase was added at 60 h, CK production increased to 12.0 mM after an additional 84 h (Figure 2D), representing a 3.4- and 4.0-fold increase compared to the production achieved by enzyme conversion with *A. niger* cellulase alone and that from the fermentation broth of *A. tubingensis*, respectively. This increase was 1.8-fold higher than the sum of the two conversions, demonstrating a synergistic effect between *A. tubingensis* fermentation and *A. niger* cellulase conversion. Therefore, the combined method was used to enhance CK production.

A direct comparison between our study and previous reports on CK production using commercial enzymes is not feasible due to differences in units of measurement. For example, in a previous study (Kim and Park, 2019), enzyme quantities were expressed in U/mL, while our study used mg/mL. However, another study on CK production using purified extracellular enzymes from *A. tubingensis* required a high concentration of 8.0 mg/mL to achieve similar CK production levels (Kim et al., 2021). In contrast, our study required only 1.25 mg/mL of *A. niger* cellulase, which is 6.4-fold lower. This suggests that the combination of fermentation and enzyme conversion is more economical, as it requires significantly less enzyme than conventional enzyme conversion methods. Additionally, this combined approach yielded a higher conversion efficiency compared to fermentation alone.

Given the improvements in both enzyme efficiency and CK yield, the combined approach has the potential to offer economic benefits in CK production. Although this study does not specifically address cost analysis, the observed synergistic effect suggests that this combined approach could be economically advantageous. The higher CK yield achieved through this method may reduce the overall cost of CK production, especially when considering the enhanced efficiency and productivity of the process. A detailed cost-benefit analysis, including enzyme and fermentation costs, will be necessary to fully assess the economic feasibility of this combined method.

### 3.3 Complementary specific activities between extracellular enzymes from *A. tubingensis* and cellulase from *A. niger* for PPD-type ginsenosides in CK production

To explain this synergistic effect, the specific activities of extracellular enzymes from *A. tubingensis* and cellulase from *A. niger* against PPD-type ginsenosides were determined (Table 1). The specific activity of extracellular enzymes from *A. tubingensis* followed the order Rb1 > Rc > CO > Rd > CMc1 > Rb2 > F2 > CMc > CY > CK, whereas that of *A. niger* cellulase followed the order Rb1 > Rd > Rb2 > Rc > CMc1 > CO > CY > F2 > CMc, with no activity observed for CK.

**FIGURE 3:**
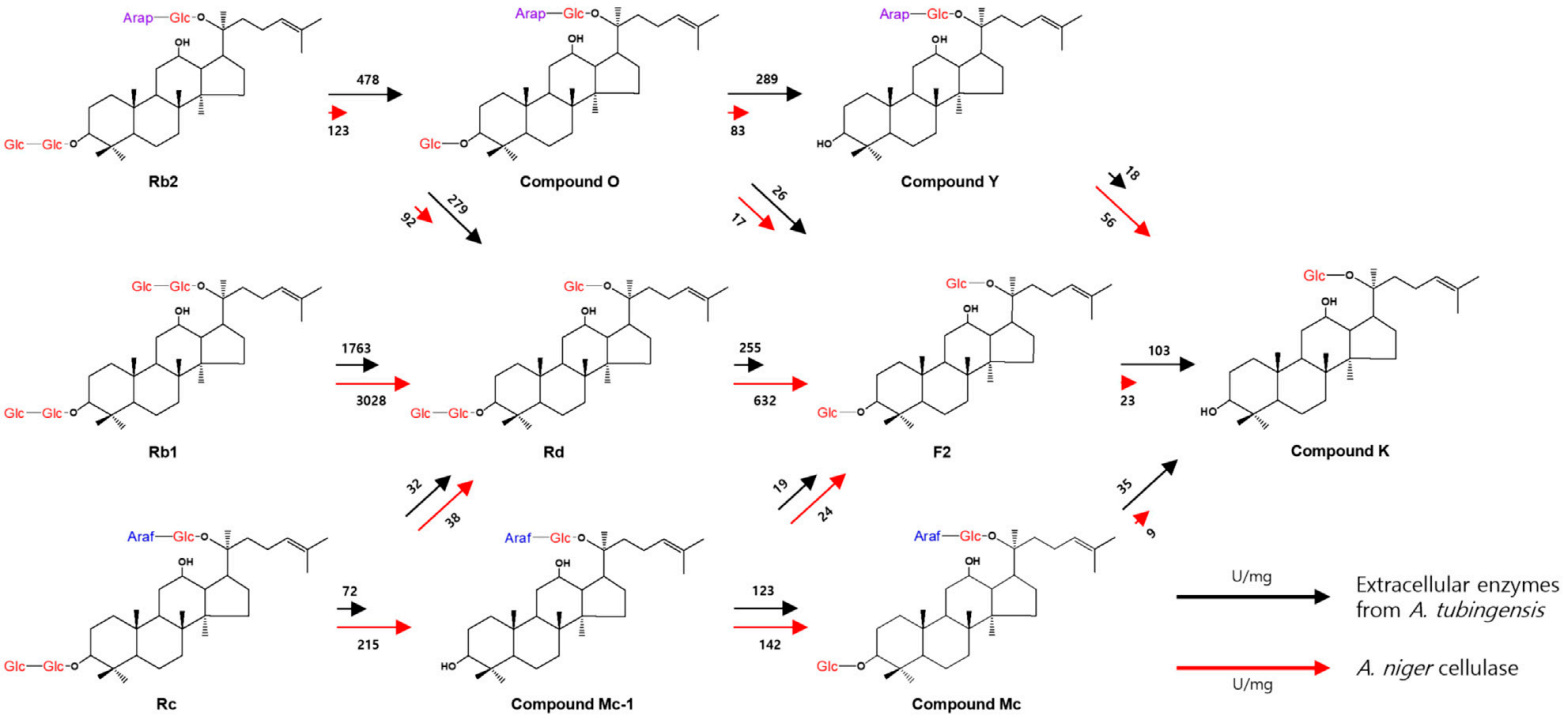
Bioconversion pathways of ginsenosides Rb1, Rb2, and Rc to CK by extracellular enzymes from *A. tubingensis* and cellulase from *A. niger*. The commercial enzyme Cellulosin AL8 is *A. niger* cellulase. The numbers located above/below the arrows and the lengths of the arrows represent the absolute specific activity values and relative specific activities of the two enzymes for each PPD-ginsenoside, respectively.

The bioconversion pathways of ginsenosides Rb1, Rb2, and Rc to CK by extracellular enzymes from *A. tubingensis* and cellulase from *A. niger* are shown in Figure 3. The activities of extracellular enzymes from *A. tubingensis* towards Rc, CO, F2, and CMc, which were converted to CMc1 and Rd, CY and F2, CK, and CK, were 3.5-, 3.5-, 4.5-, and 3.9-fold higher than that of *A. niger* cellulase, respectively (Table 1). Conversely, the activities of cellulase from *A. niger* towards Rb1, Rd, Rb2, CMc1, and CY, which were converted to Rd, F2, CO, Rd, CMc, F2, and CK, were 1.7-, 2.5-, 2.5-, 1.3-, and 3.1-fold higher than those of extracellular enzymes from *A. tubingensis*, respectively. These results indicate that the activities of the two enzymes for PPD-type ginsenosides were complementary to CK production.

### 3.4 Optimization of temperature-shift time and temperature in enzyme conversion phase for enhanced production of CK

The temperature-shift time from the cultivation phase (28°C) to the enzyme conversion phase (60°C) was varied from 48 to 96 h, with the simultaneous addition of AGE and *A. niger* cellulase at the temperature-shift time. The simultaneous addition of AGE and *A. niger* cellulase for CK production was more efficient than the separate additions. The highest CK production was observed at a temperature-shift time of 60 h (Supplementary Figure S2A). The optimal reaction temperature for the enzyme conversion phase was determined to be 60°C (Supplementary Figure S2B). When both 16 g/L AGE and 0.4 mg/mL *A. niger* cellulase were added at 60 h, enzyme conversion was performed at 60°C, resulting in the production of 5.8 mM CK after an additional 84 h (Supplementary Figure S2C).

### 3.5 Optimization of AGE and commercial enzyme concentrations during the enzyme conversion phase for enhanced production of CK

When the concentration of AGE was varied from 4 to 48 g/L at a fixed concentration of 1.0 mg/mL *A. niger* cellulase, the maximal production of CK was observed at 32 g/L AGE (Supplementary Figure S3A). As the concentration of *A. niger* cellulase was varied from 0.1 to 1.6 mg/mL with 32 g/L AGE, CK production increased with increasing enzyme concentration up to 1.0 mg/mL, beyond which it plateaued (Supplementary Figure S3B). These results indicate that the optimal ratio of AGE (32 g/L) to *A. niger* cellulase (1.0 mg/mL) was 32:1 (w/w). When the concentrations of AGE and *A. niger* cellulase at this optimal ratio were varied, the highest production of CK was observed at 40 g/L AGE and 1.25 mg/mL *A. niger* cellulase (Supplementary Figure S3C). Under these optimal conditions, CK production reached 12.0 mM in 144 h (Figure 2D), representing a 2.1-fold increase compared to the production with 16 g/L AGE and 0.4 mg/mL *A. niger* cellulase prior to optimization.

### 3.6 Enhanced production of CK by the synergistic conversion of fermentation with *A. tubingensis* and *A. niger* cellulase under optimized conditions in a flask

The reaction conditions for the synergistic conversion of fermentation with *A. tubingensis* and *A. niger* cellulase in a flask were optimized as follows: *A. tubingensis* was cultivated at 28°C for 60 h, after which 40 g/L AGE and 1.25 mg/mL cellulase from *A. niger* were added to the fermentation broth. The mixture was then incubated at 60°C for an additional 84 h. Under these optimized conditions, 12.0 mM (7.5 g/L) CK was produced from PPD-type ginsenosides in 40 g/L AGE in 144 h, with a molar yield of 74.5% and productivity of 51.5 mg/L/h (Supplementary Figure S3). For complete conversion of PPD-type ginsenosides to CK, several concentrations of AGE and *A. niger* cellulase were added to the fermentation broth of *A. tubingensis*. When 16 g/L AGE and 4.0 mg/mL *A. niger* cellulase were added to the flask, a complete conversion of 6.4 mM (6.8 g/L) of PPD-type ginsenosides to 6.4 mM (4.0 g/L) CK was achieved after 144 h, resulting in a productivity of 27.8 mg/L/h (Supplementary Table S1).

The accumulation of Rd plays a key role in the final production of CK, as Rd is converted into CK via F2 in the pathway Rb1 → Rd → F2 → CK, and F2 is directly converted into CK. The accumulation of Rd significantly slows CK production, whereas the accumulation of F2 only slightly affects it. To better understand the relationship between the accumulation of Rd and F2 in CK production, we compared the accumulation of intermediate ginsenosides in the fermentation, enzyme reaction, and combined conversion processes using 16 g/L AGE. In the fermentation process, the highest accumulated concentrations of Rd and F2 were 5.0 mM at 96 h and 1.5 mM at 120 h, respectively (Figure 1A). In the enzyme reaction, the highest accumulated concentration of F2 was 4.0 mM at 12 h (Supplementary Figure S1A). In contrast, in the combined conversion of fermentation and enzyme reaction, the highest accumulation of F2 was 3.0 mM at 72 h (Supplementary Figure S2C). These results suggest that the combined conversion process progresses more efficiently along the Rb1 → Rd → F2 → CK pathway due to the lower concentration of F2 compared to the enzyme reaction alone and the accumulation of Rd during fermentation. Therefore, the combined conversion method proved to be the most efficient for CK production.

### 3.7 Determination of temperature-shift time for enhanced production of CK in a fermenter

The pulse and continuous methods were identified as the optimal feeding methods of ginseng extract for CK production by fermentation for flask (Song et al., 2022) and fermenter experiments, respectively (Song et al., 2023). This fact was confirmed that CK production using continuous feeding of AGE in a fermenter was 3.1-fold higher than that using pulse feeding when *A. niger* cellulase was added to the fermentation broth of *A. tubingensis* (Supplementary Figure S4).

To optimize the temperature-shift time from the cultivation phase (28°C) to the enzyme conversion phase (60°C), the shift time was varied from 36 to 60 h. Cellulase from *A. niger* was added simultaneously with the temperature shift, whereas AGE was continuously supplied to the fermenter from 12 to 84 h. The CK production reached a maximum of 11.2 mM at a temperature shift time of 48 h (Table 2).

**TABLE 2:** Effect of temperature-shift timing on CK production in fermenter.

Time (h)	CK (mM)	CK (g/L)	Molar yield (%)	Productivity (mg/L/h)
36	10.3	6.4	63.8	38.0
48	11.2	7.0	69.7	41.5
54	10.5	6.6	65.6	39.1
60	10.2	6.3	63.2	37.7

### 3.8 Optimization of sucrose feeding during the cultivation phase for enhanced production of CK in a fermenter

Although glucose is a more cost-effective carbon source, its use can strongly induce carbon catabolic repression, which inhibits the production of enzymes. On the other hand, sucrose is less likely to cause carbon catabolite repression and can support more efficient fermentation for the production of CK. Similar approaches have been reported in the fermentation of *A. tubingensis* for CK production, where sucrose was selected as the carbon source instead of glucose (Song et al., 2022).

The optimal sucrose concentration added during *A. tubingensis* cultivation for enhanced CK production was evaluated by feeding sucrose as a substrate at concentrations of 6, 12, and 18 g/L from 12 to 48 h (Supplementary Figure S5). The highest CK concentration, measured at 11.2 mM, was observed at an added sucrose concentration of 12 g/L.

According to studies on the characteristics of enzyme production in fungi, high concentrations of carbon source can decrease hydrolytic enzyme activity owing to catabolic repression (Dos Reis et al., 2013). Therefore, sucrose feeding is essential for increasing hydrolytic enzyme production. The sucrose-feeding strategy was divided into two feedings: major feeding for cell growth and activity-maintaining feeding for maintenance of CK-producing activity. When a total sucrose concentration of 12 g/L was fed from 12 to 48 h, the optimal period for major feeding was determined by varying the period from 12 to 24, 36, 42, and 48 h. The highest CK production, quantified at 12.2 mM, was achieved when the major feeding period was between 12 and 36 h (Supplementary Table S2). The optimal sucrose feeding for maximal CK production was established as follows: initial addition of 20 g/L sucrose, followed by 11 g/L sucrose feeding from 12 to 36 h, and 1.0 g/L sucrose feeding from 36 to 48 h.

### 3.9 Optimization of pH, and temperature during the enzyme conversion phase for enhanced production of CK in a fermenter

The activity of hydrolytic enzymes, such as cellulase, decreased at high agitation speeds in the fermenter because of shear stress and interaction with air (Mukataka et al., 1983). Homogenized cells of *A. tubingensis* treated with a homogenizer exhibited a 1.2-fold reduction in CK productivity and conversion yield compared to non-homogenized cells (Supplementary Figure S7), suggesting that impeller stirring conditions in a fermenter may negatively affect CK-producing activity. Therefore, the agitation speed in the fermenter was set at 200 rpm during the enzyme conversion phase. Furthermore, the extended reaction time in the fermenter compared to that in the flask may be attributed to the harsh reaction conditions caused by agitation.

**FIGURE 4:**
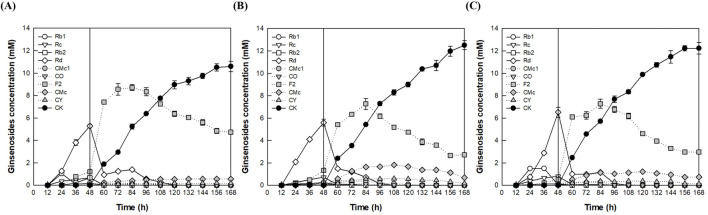
Effect of pH on CK production by the addition of *A. niger* cellulase to the fermentation broth of *A. tubingensis* in the fermenter. **(A)** pH 4.0. **(B)** pH 4.5. **(C)** pH 5.0. An AGE concentration of 40 g/L was continuously added from 12 to 84 h at a feeding rate of 0.56 g/L/h, and 1.25 mg/mL of cellulase from *A. niger* was added at 48 h. After cultivation of *A. tubingensis* at 28°C for 48 h, the reactions were performed at 60°C for an additional 120 h by varying the pH from 4.0 to 5.0.

After cultivation of *A. tubingensis* for 48 h, to determine the optimal pH during the enzyme conversion phase, the reactions were performed for an additional 120 h by varying the pH from 4.0 to 5.0 at a constant temperature of 60°C (Figure 4). CK production reached a maximum of 12.5 mM at pH 4.5, which was identified as the optimal pH for the fermenter.

**FIGURE 5:**
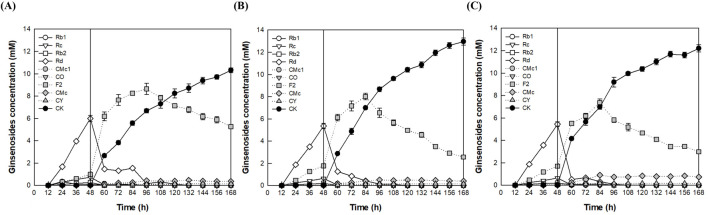
Effect of temperature on CK production by the addition of *A. niger* cellulase to the culture broth of *A. tubingensis* in the fermenter. **(A)** 50°C. **(B)** 55°C. **(C)** 60°C. An AGE concentration of 40 g/L was continuously added from 12 to 84 h at a flow rate of 0.56 g/L/h and 1.25 mg/mL *A. niger* cellulase was added at 48 h. After cultivation of *A. tubingensis* at 28°C for 48 h, the reactions were performed at pH 4.5 for an additional 120 h, by varying the temperature from 50°C to 60°C.

The temperature was varied from 50°C to 60°C while maintaining a constant pH of 4.5. After 168 h, the highest CK production was observed at 13.0 mM at 55°C, which was determined to be the optimal temperature in the fermenter (Figure 5). In the flask experiments, the optimal temperature was identified as 60°C, because CK production (12.0 mM) at this temperature after 144 h was slightly higher than that observed at 55°C (11.8 mM) (Supplementary Figure S6). However, after 168 h, CK concentration at 55°C (12.9 mM) was slightly higher than that at 60°C (12.4 mM). This difference can be attributed to a higher CK production rate at 55°C during the period from 144 h to 168 h, suggesting that enzyme stability is greater at this temperature.

### 3.10 Enhanced production of CK by the synergistic conversion of fermentation with *A. tubingensis* and *A. niger* cellulase under optimized conditions in a fermenter

The reaction conditions for the synergistic conversion of fermentation with *A. tubingensis* and *A. niger* cellulase in a fermenter were optimized as follows: *A. tubingensis* was cultivated with an initial concentration of 20 g/L sucrose at 28°C for 48 h with continuous feeding of 11 g/L sucrose from 12 to 36 h and 1 g/L sucrose from 36 to 48 h in a fermenter. An AGE concentration of 40 g/L was continuously fed from 12 to 84 h, and 1.25 mg/mL *A. niger* cellulase was added to the fermentation broth at 48 h. After this time, the reaction was conducted at pH 4.5°C and 55°C for an additional 84 h. Under the optimized conditions, a total of 13.0 mM (8.07 g/L) CK was produced in 168 h with a molar yield of 80.6% and a productivity of 48.0 mg/L/h.

When 40 g/L AGE was used, Rd and F2 accumulated up to 3.5 mM and 9.2 mM at 72 h, respectively, and CK reached 12.0 mM at 144 h in the flask (Figure 2D), while Rd and F2 accumulated up to 5.4 mM and 7.4 mM at 48 h and 84 h, respectively, and CK reached 12.2 mM at 168 h in the fermenter (Figure 5C in the revised manuscript). The higher concentrations of accumulated Rd and F2 in the flask can be attributed to the difference AGE feeding methods. In the flask, 40 g/L AGE was added once at 60 h, while in the fermenter, 40 g/L AGE was continuously added from 12 to 84 h at a flow rate of 0.56 g/L/h.

### 3.11 Comparison of CK production from PPD-type ginsenosides in ginseng extracts by fermentation without and with enzyme addition

CK has been produced from the PPD-type ginsenosides in ginseng extracts by traditional fungal fermentation. CK production by traditional fermentation was compared with that achieved by synergistic conversion of fermentation with *A. tubingensis* and commercial cellulase (Table 3). The highest previously reported concentrations of CK achieved by fermentation with *A. tubingensis* were 2.47 g/L in a flask and 3.94 g/L in a fermenter (Song et al., 2023). In contrast, CK concentrations produced by fermentation using the same strain with the addition of the enzyme reached 7.45 g/L in a flask and 8.07 g/L CK in a fermenter, with productivities of 51.5 and 48.0 mg/L/h, respectively. These results represent increases of 3.0- and 4.0-fold in the flask and 2.0- and 3.6-fold in the fermenter, respectively, compared to those achieved by fermentation without enzyme addition. Additionally, the PPD-type ginsenosides in AGE were completely converted to 4.03 g/L CK in 144 h using the combined method, with a productivity of 27.8 mg/L/h. These values were 2.1-fold higher than those obtained from fermentation using the same strain (Song et al., 2023).

**TABLE 3:** Comparison of CK production from PPD-type ginsenosides in ginseng extracts by fermentation without and with enzyme addition.

Production method	Fungus	Ginseng extract	CK (g/L)	Molar yield (%)	Productivity (mg/L/h)	Fermentation system	References
Fermentation	*Fusarium sacchari*	*Panax notoginseng* extract	0.25	24.0	1.74	Flask	Han et al. (2007)
	*Paecilomyces bainier* sp. 229	*Panax notoginseng* leaves	1.25	82.6	8.60	Fermenter	Zhou et al. (2008)
	*Ganoderma lucidum* CRC 37066	American ginseng extract	0.005	3.0	0.01	Flask	Hsu et al. (2013)
	*Aspergillus niger* KACC 46494	Korean ginseng berry extract	NC	10.3	NC	Flask	Li et al. (2016)
	*Aspergillus oryzae* KACC 40247		NC	3.4	NC		
	*Aspergillus niger* FMBS 494	Korean ginseng	0.04	66.8	0.625	Flask	Hossen et al. (2017)
	*Cordyceps sinensis*	Red ginseng extract	0.11	NC	NC	Fermenter	Bae et al. (2011)
	*Aspergillus tubingensis* KCTC 14166	American ginseng extract	2.47^a^	64.3	17.1	Flask	Song et al. (2022)
	*Aspergillus tubingensis* KCTC 14166	American ginseng extract	1.91^b^	100	13.2	Fermenter	Song et al. (2023)
			3.94^b^	82.1	27.4		
Fermentation with enzyme addition	*Aspergillus tubingensis* KCTC 14166	American ginseng extract	4.03^c^	100	27.8	Flask	This study
			7.45^d^	74.5	51.5	Flask	
			8.07	80.6	48.0	Fermenter	

NC, not calculated.

^a^ AGE, at 8 g/L were added twice at 48 and 60 h.

^b^ AGE, at 8 g/L and 20 g/L were continuously added from 12 to 132 h.

^c^ AGE, at 16 g/L and *A. niger* cellulase at 4.0 mg/mL were reacted in 60°C.

^d^ AGE, at 40 g/L and *A. niger* cellulase at 1.25 mg/mL were reacted in 60°C.

*Paecilomyces bainier* exhibited the highest previously reported CK concentration (1.25 g/L) and productivity (8.6 mg/L/h) using another fungal strain (Zhou et al., 2008), which were 6.5- and 5.6-fold lower than those achieved with the combined method, respectively. These findings indicate that the combination of *A. tubingensis* fermentation and *A. niger* cellulase conversion is an effective strategy to enhance the biotransformation of PPD-type ginsenosides into CK.

## 4 Conclusion

CK production from ginseng extract by fermentation has limitations such as low concentration and productivity. The combination of fermentation and enzyme conversion provides a viable solution. The key factors influencing enzyme conversion, including sucrose feeding during the cultivation phase, timing of fermentation and enzyme conversion, concentrations of AGE and enzymes, and temperature and pH during the enzyme conversion phase, were optimized. Under the optimized conditions, CK concentration and productivity increased due to the synergistic effect of the complementary activities of enzymes produced by *A. tubingensis* and commercial cellulase from *A. niger* compared to traditional fermentation. To the best of our knowledge, this is the first study to combine fermentation with enzyme conversion to enhance CK production.

## Data Availability

The raw data supporting the conclusions of this article will be made available by the authors, without undue reservation.

## Abbreviations

AGE, American ginseng extract; CK, compound K; CMc, compound Mc; CMc1, compound Mc1; CO, compound O; CY, compound Y; PPD, protopanaxadiol.
