# Supplementary material for: Enhanced production of ginsenoside compound K by synergistic conversion of fermentation with Aspergillus tubingensis and commercial cellulase
Source: Front Bioeng Biotechnol. 2025 Jan 8;12:1538031. doi: 10.3389/fbioe.2024.1538031 (PMC11750783; doi:10.3389/fbioe.2024.1538031)
Supplement: Supplementary file 1 [file Presentation1.pptx]

## Slide 1
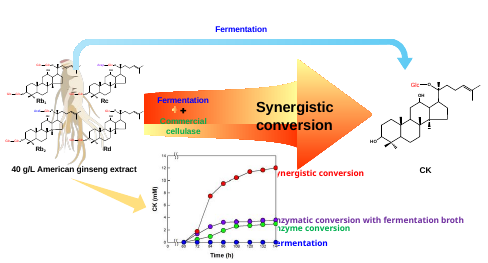

Fermentation
Rb1
Rc
Rb2
Rd
Fermentation
Commercial cellulase
Synergistic conversion
CK
40 g/L American ginseng extract
Synergistic conversion
Enzymatic conversion with fermentation broth
Enzyme conversion
Fermentation
