# Supplementary material for: Enhanced production of ginsenoside compound K by synergistic conversion of fermentation with Aspergillus tubingensis and commercial cellulase
Source: Front Bioeng Biotechnol. 2025 Jan 8;12:1538031. doi: 10.3389/fbioe.2024.1538031 (PMC11750783; doi:10.3389/fbioe.2024.1538031)
Supplement: Supplementary file 2 [file DataSheet1.docx]

Supplementary Material

# TABLE S1 CK production by varying the concentrations of *A. niger* cellulase and AGE for complete conversion of PPD-type ginsenosides to CK.

| AGE (g/L) | *A. niger* cellulase (mg/mL) | CK (mM) | CK (g/L) | Molar yield (%) | Productivity (mg/L/h) |
| --- | --- | --- | --- | --- | --- |
| 12 | 1.0 | 4.2 | 2.6 | 86.5 | 18.0 |
|  | 2.0 | 4.4 | 2.7 | 91.3 | 19.1 |
|  | 4.0 | 4.8 | 3.0 | 100.0 | 20.9 |
| 16 | 1.0 | 5.5 | 3.4 | 86.1 | 23.9 |
|  | 2.0 | 5.8 | 3.6 | 89.9 | 25.0 |
|  | 4.0 | 6.4 | 4.0 | 100.0 | 27.8 |

Temperature was changed from 28°C to 60°C, and AGE and *A. niger* cellulase were added at 60 h.

# TABLE S2 Effect of sucrose feeding period during the cultivation phase on CK production by adding *A. niger* cellulase in a fermenter.

| Major feeding | | Activity-maintaining feeding*^a^* | | CK  (mM) | CK  (g/L) | Molar yield (%) | Productivity (mg/L/h) |
| --- | --- | --- | --- | --- | --- | --- | --- |
| Feeding period | Sucrose (g/L) | Feeding period | Sucrose (g/L) |  |  |  |  |
| 12 ~ 24 h | 10.0 | 24 ~ 48 h | 2.0 | 10.4 | 6.5 | 65.0 | 38.7 |
| 12 ~ 36 h | 11.0 | 36 ~ 48 h | 1.0 | 12.2 | 7.6 | 76.1 | 45.4 |
| 12 ~ 42 h | 11.5 | 42 ~ 48 h | 0.5 | 11.8 | 7.3 | 73.2 | 43.7 |
| 12 ~ 48 h | 12.0 |  |  | 11.2 | 7.0 | 69.7 | 41.5 |

*^a^*The activity-maintaining feeding rate of sucrose was 0.083 g/L/h.

**(A)**

**(B)**

**(C)**

**FIGURE S1**

CK production from PPD-type ginsenosides in AGE via fermentation and/or enzyme conversion. (A) CK production by enzymatic conversion using *A. niger* cellulase*.* The reaction was performed at 60 ℃ in 50 mM citrate/phosphate buffer (pH 5.0) containing 16 g/L AGE and 0.5 mg/mL *A. niger* cellulase for 60 h. (B) CK production via enzyme conversion after fermentation without enzyme addition*.* *A. tubingensis* was cultured at 28 ℃ for 60 h, 16 g/L AGE was added at 60 h, and the reaction was performed at 60 ℃ for an additional 84 h. (C) CK production by enzyme conversion after fermentation with enzyme addition*. A. tubingensis* was cultured at 28℃ for 60 h, 16 g/L AGE and 0.5 mg/mL *A. niger* cellulase were added at 60 h, and the reaction was performed at 60 ℃ for an additional 84 h.

**(A)**

**(B)**

**(C)**

**FIGURE S2**

Effects of temperature-shift time and temperature during the enzyme conversion phase on CK production. (A) Effect of temperature-shift time. At each temperature shift time, 0.4 mg/mL *A. niger* cellulase and 16 g/L AGE were added to the cultivation broth. (B) Effect of temperature. After the cultivation of *A. tubingensis* at 28 °C for 60 h, 0.4 mg/mL *A. niger* cellulase and 16 g/L AGE were added to the fermentation broth at 60 h. (C) Time-course reactions of CK production by adding *A. niger* cellulase to the fermentation broth of *A. tubingensis* at the optimized temperature-shift time and temperature. After the cultivation of *A. tubingensis* at 28 °C for 60 h, 16 g/L AGE and 0.4 mg/mL *A. niger* cellulase were added at 60 h, and the reaction was performed at 60 °C for an additional 84 h.

**(A)**

**(B)**

**(C)**

**FIGURE S3**

Effects of AGE and *A. niger* cellulase concentrations on CK production during the enzyme conversion phase. After the cultivation of *A. tubingensis* at 28 °C for 60 h, AGE and *A. niger* cellulase were added to the culture broth at 60 h, and the reactions were performed at 60°C for an additional 84 h. (A) Effect of AGE concentration. The reactions were performed by varying the AGE concentration from 4 to 48 g/L with 1.0 mg/mL *A. niger* cellulase. (B) Effect of *A. niger* cellulase concentration. The reactions were performed by varying the concentration of *A. niger* cellulase from 0.1 to 1.5 mg/mL with 32 g/L AGE. (C) Effect of AGE and *A. niger* cellulase concentrations at the optimal ratio. The reactions were performed by varying the concentrations of AGE and *A. niger* cellulase at an optimal ratio of 32:1 (w/w) from 8 and 0.25 to 64 g/L and 2.0 mg/mL.

**(A)**

**(B)**

**FIGURE S4**

CK production by pulse and continuous AGE feeding in a fermenter. (A) CK production after pulse AGE feeding. AGE at 40 g/L with 1.25 mg/mL *A. niger* cellulase was added at 60 h after the cultivation of *A. tubingensis* at 28 °C for 60 h. (B) CK production by continuous AGE feeding. AGE at 40 g/L was continuously added from 12 to 84 h at a feeding rate of 0.56 g/L/h, while 1.25 mg/mL *A. niger* cellulase was added at 48 h after the cultivation of *A. tubingensis* at 28℃ for 48 h.

**(A)**

**(B)**

**(C)**

**FIGURE S5**

Effect of added sucrose concentration on CK production during the cultivation phase in a fermenter. (A) Feeding with 6 g/L sucrose. Sucrose at 6 g/L was added from 12 to 48 h at a feeding rate of 0.17 g/L/h (B) Feeding of 12 g/L sucrose. Sucrose at 12 g/L was added from 12 to 48 h at a feeding rate of 0.33 g/L/h. (C) Feeding with 18 g/L sucrose. Sucrose at 18 g/L was added from 12 to 48 h at a feeding rate of 0.50 g/L/h. Additionally, 20 g/L sucrose was initially added, *A. niger* cellulase at 1.25 mg/mL was added at 48 h, and 40 g/L AGE was continuously added from 12 to 84 h at a feeding rate of 0.56 g/L/h.

**(A)**

**(B)**

**FIGURE S6**

CK production in flasks at 55 °C and 60 °C by adding *A. niger* cellulase to the fermentation broth of *A. tubingensis*. After cultivation of *A. tubingensis* at 28 °C for 60 h, 40 g/L AGE, and 1.25 mg/mL *A. niger* cellulase were added at 60 h, and the reactions were performed for an additional 108 h. (A) CK production at 55 °C. (B) CK production at 60 °C.

**(A)**

**(B)**

**FIGURE S7**

Biotransformation of PPD-type ginsenosides in AGE to CK by homogenized and non-homogenized *A. tubingensis* in flasks. After cultivation of *A. tubingensis* at 28 °C for 60 h, 40 g/L AGE and 1.25 mg/mL *A. niger* cellulase were added at 60 h, and the enzyme reactions were performed at 60 °C for an additional 84 h. (A) CK production by homogenized *A. tubingensis*. *A. tubingensis* was grown and harvested at 60 h, the harvested *A. tubingensis* was treated with a homogenizer before used in CK production. (B) CK production by non-homogenized *A. tubingensis*.
